# Study on the Wear Resistance of 6061 Aluminum Alloy Bipolar Plasma Electrolytic Oxidation Ceramic Coating by the Addition of K_2_ZrF_6_

**DOI:** 10.3390/ma18132962

**Published:** 2025-06-23

**Authors:** Rui Tong, Shiquan Zhou, Hongtao Li, Xiang Tao, Jian Chen

**Affiliations:** College of Materials Science and Engineering, Nanjing Tech University, Nanjing 211816, China; 202261203248@njtech.edu.cn (R.T.); 202261203164@njtech.edu.cn (S.Z.); 202261203226@njtech.edu.cn (X.T.); 202361203196@njtech.edu.cn (J.C.)

**Keywords:** aluminum alloy, K_2_ZrF_6_, plasma electrolytic oxidation, wear resistance

## Abstract

A plasma electrolytic oxidation (PEO) coating was produced on 6061 aluminum alloy within a silicate-containing electrolyte using a bipolar pulsed power supply. The impact of K_2_ZrF_6_ addition on the wear resistance of the coating was investigated. The phase composition, surface morphology, and elemental distribution of the coatings were assessed by means of X-ray diffraction (XRD), energy-dispersive spectroscopy (EDS), and scanning electron microscopy (SEM). Experimental data revealed that the growth rate of the coating increased by 37.3% compared to that without K_2_ZrF_6_; the addition of K_2_ZrF_6_ favored the formation of mullite and enhanced the coating densification; it also improved the breakdown voltage of the coating, which increased by 46.0% compared to that without K_2_ZrF_6_; and it also demonstrated excellent abrasion resistance, with a reduction of 41.8% in the weight of the abrasion.

## 1. Introduction

Due to their favorable strength-to-weight ratio, aluminum alloys find extensive use in automotive and transportation components. However, their low hardness and poor wear resistance often limit their applicability [1,2]. Micro-arc oxidation (MAO), alternatively termed PEO, enables the formation of an adherent ceramic coating on aluminum substrates, markedly enhancing their wear resistance, corrosion resistance, and adhesion strength [3,4,5]. The performance of the ceramic coating is primarily influenced by the electrolyte composition and electrical parameters [6,7].

Soft plasma discharges have received much attention in recent years. In conventional plasma electrolytic oxidation, oxide generation is known to be related to the anodic polarization; however, the rate and quality of oxide generation were found to increase when negative pulses were introduced [8,9]. In particular, during PEO processing of aluminum alloys, a distinct soft plasma discharge mode is activated when the cathodic current density surpasses its anodic counterpart. This discharge mode significantly enhances both the deposition rate and coating quality [10,11]. The appearance of soft plasma is often accompanied by a uniform distribution of sparks, a reduction in the intensity of the discharge and a reduction in acoustic emission [11,12,13]. An obvious increase in the content of α-Al_2_O_3_ was found in the ceramic layers formed in the soft plasma, and this formation of α-Al_2_O_3_ in the soft plasma is one of the most interesting phenomena in MAO research [13].

However, despite the enhanced coating density achievable through soft plasma processing of aluminum surfaces, the coating hardness depends only on the percentage of α-Al_2_O_3_ in the coating, which determines the maximum value of the coating hardness [14]. Multiple studies have aimed at toughening the coating or increasing the hardness of the coatings by adding appropriate nanoparticles to the electrolyte to obtain a ceramic layer with the corresponding oxides. Amin Hakimizad [15] found that using W-doped silica-based solution in bipolar mode reduces the porosity and at the same time generates high-hardness coatings. D.V. Mashtalyar et al. [16] prepared coatings with better mechanical properties and higher microhardness by adding titanium nitride and sodium dodecyl sulfate to the electrolyte. Zhang et al. [17] found that PEO coatings incorporating nanoparticles had a smaller pore size and better wear and corrosion resistance. Matykina et al. [18] prepared coatings with wear resistance by adding zirconium dioxide to the electrolyte. Zirconia-toughened alumina has enhanced properties including hardness, wear resistance, and most importantly, higher fracture toughness than monolithic Al_2_O_3_ [19,20,21]. However, higher concentrations of NP require the use of surfactants, which is not favorable for industrial applications [22].

Several authors have confirmed the benign effect of using K_2_ZrF_6_ additives in PEO coatings. For example, Zhang et al. [23] observed an increase in the formation rate and uniformity of MAO coatings on aluminum alloys with the addition of K_2_ZrF_6_. By K_2_ZrF_6_ addition, the current was increased, which resulted in the maximum solid melt deposition and formation rate. Further reports showed that the F^−^ provided by K_2_ZrF_6_ led to an increase in homogeneity and made the inner layer denser [24,25].

The current study aims to investigate the influence of K_2_ZrF_6_ doping on the wear resistance of PEO coatings during soft plasma discharge. This investigation evaluates the impact of K_2_ZrF_6_ doping on the growth kinetics, breakdown voltage, and wear resistance of coatings while also introducing a wear reduction mechanism.

## 2. Methods and Materials

### 2.1. Experiment Preparation

A 6061 aluminum alloy cylinder (Φ 35 × 3 mm) with a composition of 0.8–1.2% Mg, 0.4–0.8% Si, 0.7% Fe, 0.15–0.4% Cu, 0.25% Zn, 0.04–0.35% Cr, 0.15% Ti, 0.15% Mn and balanced Al was utilized as the base material. Prior to PEO processing, the specimens were mechanically polished by the use of SiC abrasives (up to 2000 grit), followed by ethanol ultrasonic cleaning (15 min) before being re-cleaned using a large amount of deionized water, blown dry and set aside.

### 2.2. Plasma Electrolytic Oxidation

The oxidation process was conducted in an aqueous electrolyte system with a Na_2_SiO_3_ (AR, Sinopharm Chemical Reagent Co., Ltd., Shanghai, China) to (NaPO_3_)_6_ (AR, Sinopharm Chemical Reagent Co., Ltd., Shanghai, China) ratio of 2:1, maintained at 20 °C via a recirculating cooling circulation system. The sample, serving as the anode, was connected to a pulsed power supply opposite a stainless steel cylindrical cathode. The electrical parameters were configured as follows: current density (6 A/dm^2^ cathodic, 7.5 A/dm^2^ anodic) at a 500 Hz frequency with a 20% duty cycle and a plasma electrolytic oxidation treatment duration of 40 min. K_2_ZrF_6_ (AR, Shanghai Aladdin Biochemical Technology Co., Ltd., Shanghai, China) and citric acid (CA) were sequentially added to the electrolyte for three experiments, S1 (0 g/L K_2_ZrF_6_), S2 (5 g/L K_2_ZrF_6_), and S3 (5 g/L K_2_ZrF_6_ + CA). The corresponding relationship between the number of samples and the amount of K_2_ZrF_6_ added in the experiment is shown in Table 1.

### 2.3. Coating Performance Test Methods

Coating thickness was measured by means of an eddy current thickness gauge at five randomly selected points on each side of the specimen (accuracy 0.1 μm), the average of which indicated the thickness of the coating (FMP20, Helmut Fischer GmbH, Waldkirch, Germany). The surface roughness of plasma electrolytic oxide coatings was tested utilizing a roughness meter (TR-210, Helmut Fischer GmbH, Waldkirch, Germany). Phase analysis was performed by use of an X-ray diffractometer (XRD, D/Max-2400, Rigaku Corporation, Tokyo, Japan) operating in grazing-incidence mode (3° incidence angle) with a 10–90° range, 0.02° step size, and 8°/min scanning rate. Surface morphology and elemental composition were examined with a scanning electron microscope (SEM, JSM-IT500, JEOL Ltd., Tokyo, Japan) coupled with an X-ray energy spectrometer (EDS, X-ACT, JEOL Ltd., Japan, Tokyo, Tokyo). The cross-sectional hardness of the coating was measured using a microhardness tester (HVT-1000, Jinan Hansen Precision Instruments, Jinan, China). The wear resistance of the prepared plasma electrolytic oxidation coating was characterized by a friction tester (XLGT200, Xi’an University of Technology, Xian, China). The friction coefficient during the process was recorded and the mass wear of the coating was measured by a balance. The friction test parameters are shown in Table 2.

## 3. Results and Discussion

### 3.1. Effect of K_2_ZrF_6_ Addition on the Time-Dependent Voltage Profile

**Figure 1:**
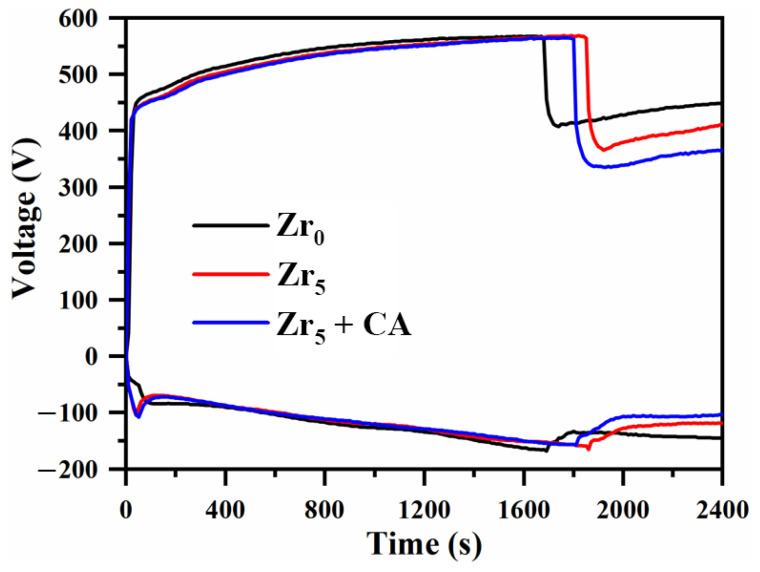
Time-dependent voltage profile during PEO of aluminum alloy with K_2_ZrF_6_ addition.

Bipolar plasma electrolytic oxidation of 6061 aluminum alloy was carried out in an Na_2_SO_3_-K_2_ZrF_6_ electrolyte system. The time-dependent voltage profile during plasma discharge of the aluminum alloy with K_2_ZrF_6_ is presented in Figure 1. As evident from the figure, the overall trend is the same when the positive voltage rises under the three parameters, and the difference is the time of appearance of the soft plasma discharge. After treatment of 5 g/L K_2_ZrF_6_, the appearance of the soft plasma discharge was delayed by about 3 min, and the minimum voltage was reduced by 42 V compared with it. The addition of citric acid (CA) after the addition of K_2_ZrF_6_ delayed the appearance of the soft plasma discharge by about 1 min, and the minimum voltage was 24 V lower than that with only K_2_ZrF_6_. The change in the time–voltage profile affects the amount of energy produced by the discharge in the plasma electrolytic oxidation, so that the growth rate of the coatings and the quality of the film formation are changed.

**Figure 2:**
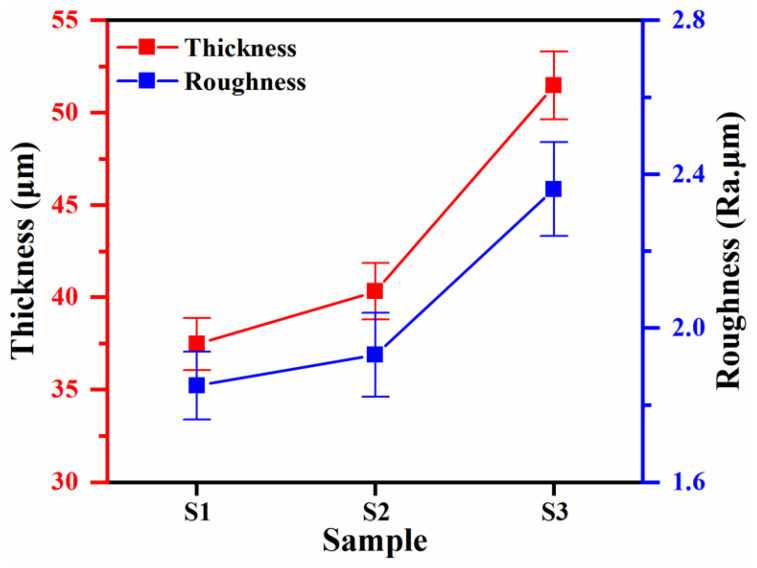
Correlation between coating thickness and roughness of PEO aluminum alloy with K_2_ZrF_6_ addition.

The relationship between the thickness and roughness of aluminum alloy plasma electrolytic oxidation coatings under the conditions of K_2_ZrF_6_ addition is shown in Figure 2. From the figure, it can be seen that the thickness and roughness of the coating have changed, the coating thickness of the S2 parameter has only increased by 7.6% relative to S1, and the change in roughness has increased by 4.3%, which is not a very obvious trend, which indicates that the content of K_2_ZrF_6_ in the coating is low. Compared to S1, the S3 parameters yielded a plasma electrolytic oxidized coating with 37.3% increased thickness and 27.6% increased roughness. This was due to the low solubility of K_2_ZrF_6_ when citric acid (CA) was not added and the electrolyte pH was tested to be 13.20 using a pH meter. When the appropriate amount of citric acid was added, the pH of the electrolyte was lowered to about 7.7, which provided a near-neutral environment, promoted the dissolution of K_2_ZrF_6_, and facilitated better participation of K_2_ZrF_6_ in the reaction of the coating. However, based on Figure 1, the soft plasma appeared later in the S3 parameter than in the S1 parameter, and the previous results proved that the growth rate before [26] soft plasma appeared was higher than after, which may be one of the reasons for the coating thickening. Based on component voltage theory by Zhang et al. [27], under spark discharge conditions, the majority of the positive voltage is distributed across the inner barrier layer. This potential corresponds to the critical positive voltage observed at the inflection point in the V-t curve (current-derived), termed the inner-barrier-layer positive voltage. The remaining voltage potential (outer-layer voltage) facilitates continuous outer-layer growth. Therefore, the micro-arc discharge stage is mainly aimed at the growth of the coating and the soft plasma discharge stage acts on the densification of the coating.

### 3.2. Phase Composition and Microscopic Morphology of Coatings

**Figure 3:**
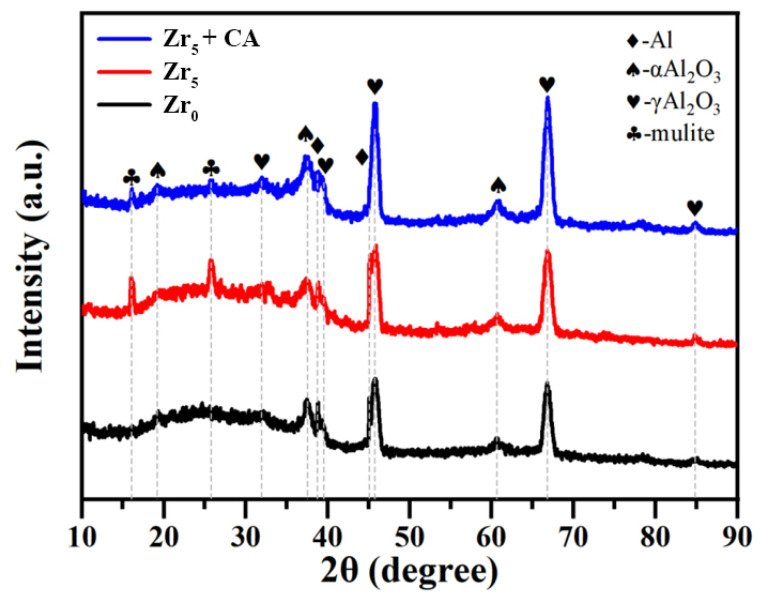
XRD analysis of aluminum alloy PEO coatings under the conditions of K_2_ZrF_6_ addition.

The XRD pattern of 6061 aluminum alloy PEO coating under the condition of K_2_ZrF_6_ addition is shown in Figure 3; the PEO coatings prepared before the addition of K_2_ZrF_6_ only have diffraction peaks of α-Al_2_O_3_ and γ-Al_2_O_3_, and there are no extra stray peaks. Among them, the content of γ-Al_2_O_3_ was lower compared with that under other parameters. When 5 g/L of K_2_ZrF_6_ was added to the electrolyte, two stronger diffraction peaks of a mullite phase appeared, which indicated that the addition of K_2_ZrF_6_ favored the generation of the mullite phase. The increasing mullite (3Al_2_O_3_-2SiO_2_) confirms the active involvement of electrolyte-derived silicon in the plasma electrolytic oxidation process. The lack of detectable SiO_2_ suggests either the generation of amorphous silicon oxide or complete reaction with alumina to produce mullite through high-temperature sintering during discharge events. Mullite has excellent physical, chemical, and mechanical properties [14] and is one of the extremely important crystalline phases of ceramic materials, which can be widely used in the field of wear-resistant materials. When citric acid was added, the diffraction peaks of α-Al_2_O_3_ and γ-Al_2_O_3_ became stronger, but the diffraction peaks of mullite weakened. According to a literature review, the generation of a mullite phase is more favorable in an alkaline environment [28].

**Figure 4:**
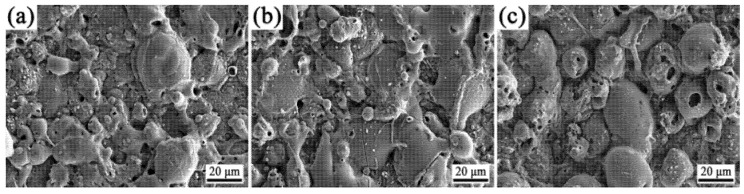
Surface morphology of PEO coatings under the conditions of K2ZrF6 addition; (**a**) 0 g/L, (**b**) 5 g/L, (**c**) 5 g/L + CA.

The surface morphology of the PEO coating (Figure 4) demonstrated that K_2_ZrF_6_ addition produced PEO coatings with stable surface morphologies (Figure 4a,b), preserving the discharge channel architecture established during the soft plasma regime. After the addition of citric acid, the surface of the coating has a more “coral“-like morphology, and the surface of the “coral“-like morphology [29] has more pores, which may be the discharge channel yielded by the volcanic eruption in the micro-arc discharge stage, and provide the conditions for the subsequent soft plasma discharge. As displayed in Figure 2, the coatings with S3 parameters have higher thickness and roughness, more discharge channels are favorable for the film formation rate in the soft plasma stage, and the increase in the coating growth rate also results in a larger roughness.

**Figure 5:**
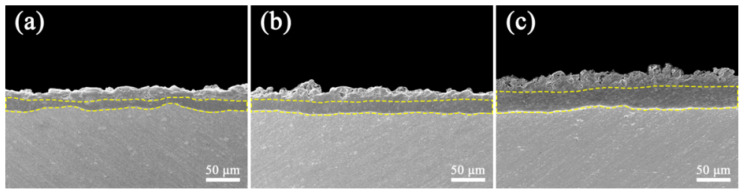
Cross-sectional morphology of PEO coatings with K_2_ZrF_6_ addition; (**a**) 0 g/L, (**b**) 5 g/L, (**c**) 5 g/L + CA.

Cross-sectional morphological analysis (Figure 5) revealed the microstructure of K_2_ZrF_6-_modified PEO coating. The overall morphology of the coating cross-section is relatively dense, but the percentage of the dense layer inside the coating is not high (about 45%) and the film formation of the dense layer is not uniform. The cross-sectional morphology of the coatings with the addition of K_2_ZrF_6_ (Figure 5b) is overall denser and has a relatively high percentage of dense layers (about 50%), with the outer layer being sparser compared to that without K_2_ZrF_6_. When citric acid was added, the PEO coating thickness predominantly increased, the inner coating was denser and had the highest percentage of dense layers (about 60%), but there was also the disadvantage that the outer layer of the coating was looser, contributing to an enhancement of the coating surface roughness (Figure 2).

### 3.3. EDS Analysis and Elemental Maps of Coated Cross-Sections

**Figure 6:**
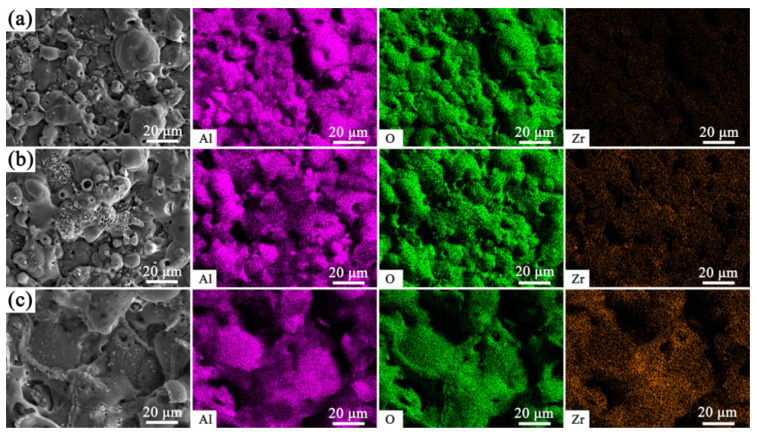
Surface EDS element distribution of PEO coatings with K_2_ZrF_6_ addition; (**a**) 0 g/L, (**b**) 5 g/L, (**c**) 5 g/L + CA.

The surface EDS elemental distribution of the PEO coatings under the conditions of K_2_ZrF_6_ addition is displayed in Figure 6. The distribution of zirconium elements in the coating only gradually becomes obvious when 5 g/L of K_2_ZrF_6_ and citric acid are added. An analysis of the elemental content of the PEO coating surfaces under these three different experimental parameters (Table 3) shows that following K_2_ZrF_6_ addition, there is an evident reduction in the aluminum element of the coatings, and in its place, there is an increase in the silicon element, which creates the conditions for the formation of mullite peaks. However, when only K_2_ZrF_6_ is added, the content of zirconium in the coating is only 0.08%, indicating that K_2_ZrF_6_ does not act directly on the coating but plays a catalytic role. When citric acid was added, the amount of elemental zirconium in the coating increased, but only to 1.26%, which could be the reason why zirconium oxide crystals were not detected.

**Figure 7:**
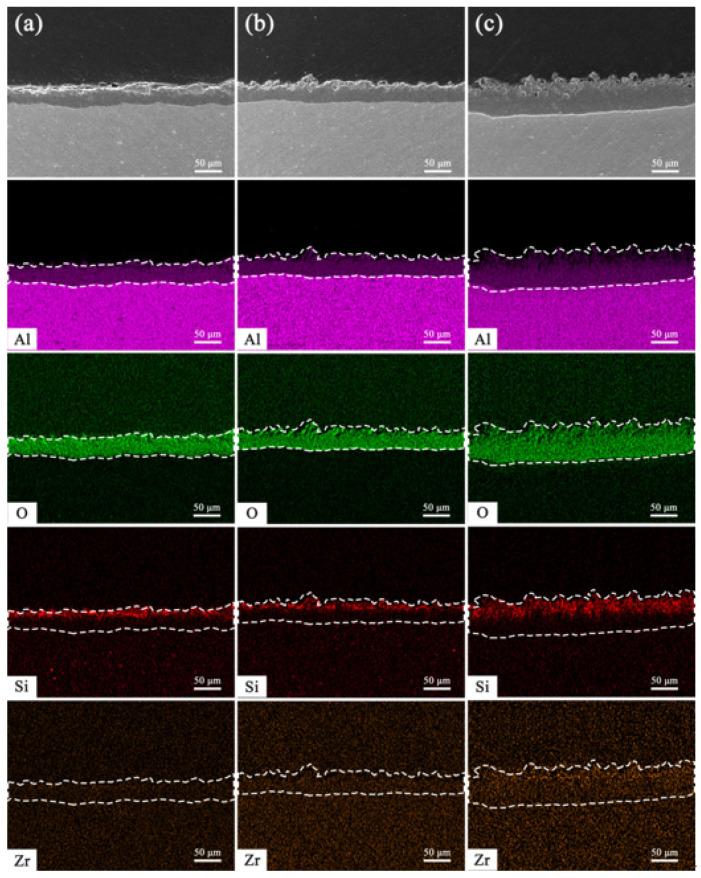
Cross-sectional EDS element distribution analysis of PEO coatings with K_2_ZrF_6_ addition; (**a**) 0 g/L, (**b**) 5 g/L, (**c**) 5 g/L + CA.

**Figure 8:**
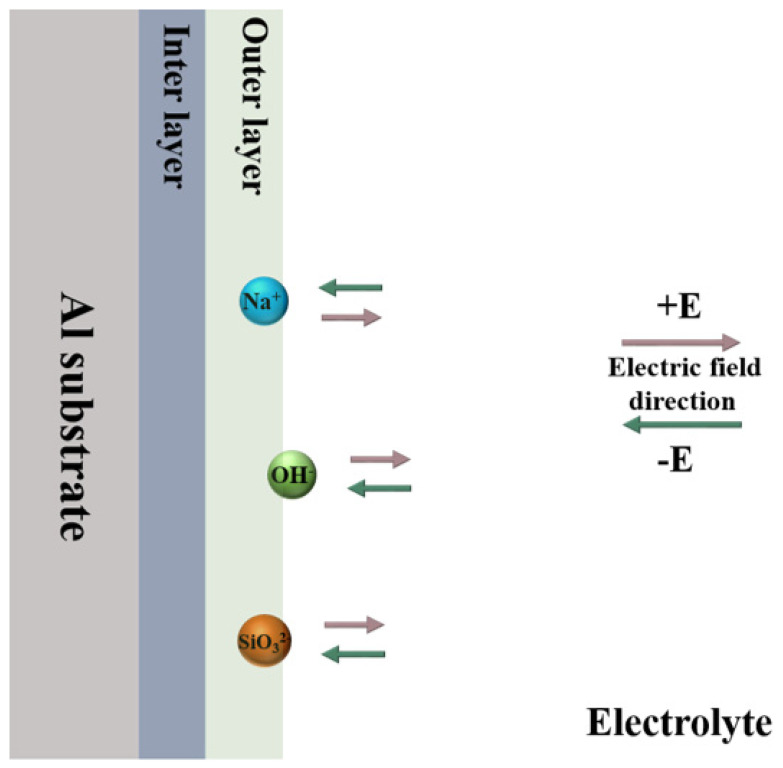
Schematic diagram of the movement of anions and cations in the electric field during plasma electrolytic oxidation in bipolar mode.

Figure 7 presents the cross-sectional EDS elemental distribution of the PEO coatings under the condition of K_2_ZrF_6_ addition. Accordingly, the distribution of silicon elements is still mainly present in the outer layer of the coating, which is the result of the generation of the soft plasma discharge phase. The silicon element in Figure 7c has an obvious extension to the inner layer, and the reason for this result may be that the PEO coating prepared with the addition of citric acid suffers from a sparser outer layer relative to the other two coatings, which is conducive to the entry of silicon into the inner part of the coating. Due to the low content of zirconium elements in the coating, the distribution state in the coating is not significant. However, a small distribution of zirconium elements is still revealed in Figure 7c, and it can be found that the distribution state of zirconium elements and silicon elements is basically the same, mainly distributed in the outer layer of the coating. This explains the fact that during the soft plasma discharge stage, the main elements involved in the reaction of the PEO coating are aluminum and oxygen. Figure 8 shows a schematic diagram of the movement of anions and cations in the electric field during plasma electrolytic oxidation in bipolar mode. As can be seen in the figure, there are two electric fields during the plasma electrolytic oxidation process. When the positive electric field begins to work, the anions in the electrolyte begin to move toward the anode and the cations toward the cathode; when the negative electric field begins to operate, the opposite is true. Therefore, when the negative current density is increased, the strength of the negative electric field becomes stronger, and the process of anion movement toward the anode is weakened by the electric field force. This results in the Si element in SiO_3_^2−^ only aggregating in the outer layer of the PEO coating.

**Figure 9:**
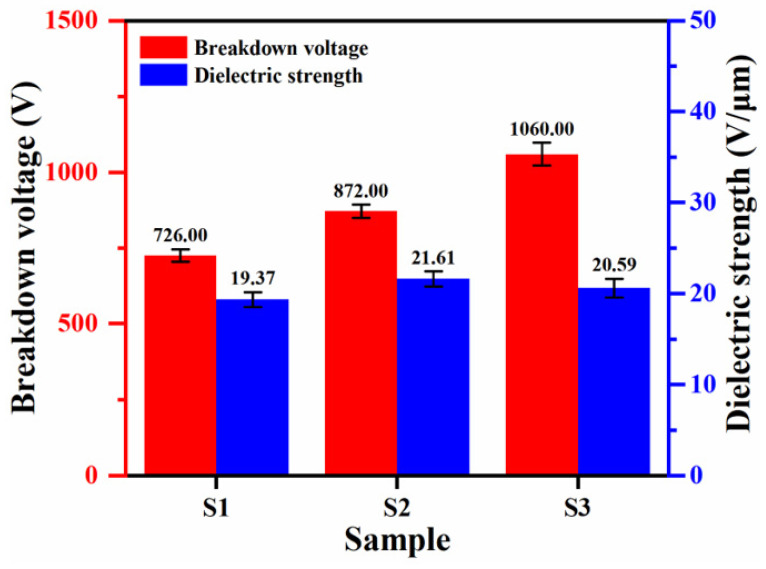
Breakdown voltage and insulation strength of PEO coatings under the conditions of K_2_ZrF_6_ addition.

Figure 9 shows the breakdown voltage and dielectric strength of the PEO coatings with K_2_ZrF_6_ addition. The result demonstrated that K_2_ZrF_6_ improves the insulating properties of the coatings, and when only K_2_ZrF_6_ is added (S2), the breakdown voltage is increased by 20.1% relative to S1. As can be seen in Figure 5b, the coating under the S2 parameter exhibits a more compact inner layer and has a higher percentage of dense layers than that under the S1 parameter. As a result, the breakdown voltage (dielectric strength) per unit thickness is higher for the S2 parameter than for S1. However, its insulation strength did not improve relative to S2 but decreased by 4.7%. As can be seen in Figure 5c, although the percentage of dense layers of the PEO coating prepared by S3 is higher than that of S2, the outer layer of the coating exhibits a more loose state, so the overall insulation strength of the coating is pulled down.

### 3.4. Tribological Behavior of the Coating

**Figure 10:**
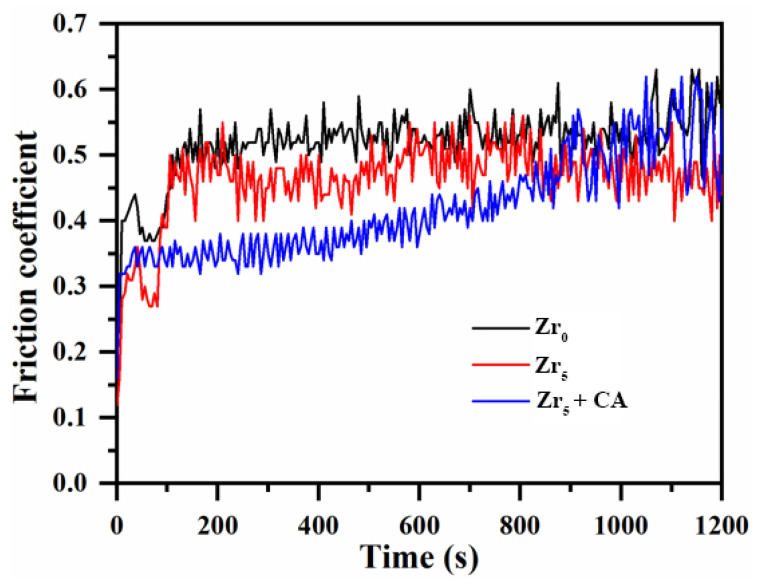
Friction coefficient and time(s) of PEO coating under the condition of K_2_ZrF_6_ addition.

Figure 10 presents the evolution of the friction coefficient for K_2_ZrF_6_-modified PEO coatings as a function of time. As evident from the figure, the PEO coatings in both bipolar modes exhibit smoother friction profiles. Without the addition of K_2_ZrF_6_, the initial coefficient of friction was relatively low, and the coefficient of friction began to increase from 0.4 to 0.5 after a duration of about 2 min, and then the coefficient of friction remained around 0.5 until the end. When K_2_ZrF_6_ is added, the initial friction curve coefficient only fluctuates up and down to 0.3, and the coefficient of friction also rises to 0.5 before entering the next wear stage. However, this stage is not very stable; the fluctuation of the friction coefficient is relatively large, and the mullite diffraction peaks could be clearly evaluated through the XRD pattern (Figure 3). Although the mullite phase has excellent wear-resistant properties in the coating, the non-uniform distribution of the mullite phase in the coating leads to the instability of the friction curves, and this state lasts until the end. When citric acid was added to the electrolyte, the initial coefficient of friction was 0.35. Unlike the other two friction curves, the friction curve at this parameter does not rise abruptly but in a slower and smoother trend. This may be due to the thicker loose layer of the coating, which slowly and continuously increases the wear rate during the wear process, resulting in the coefficient of friction also being in such a state.

**Figure 11:**
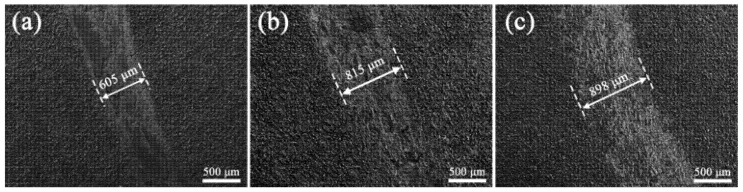
Wear scar morphology of PEO coatings with K_2_ZrF_6_ addition; (**a**) 0 g/L, (**b**) 5 g/L, (**c**) 5 g/L + CA.

**Figure 12:**
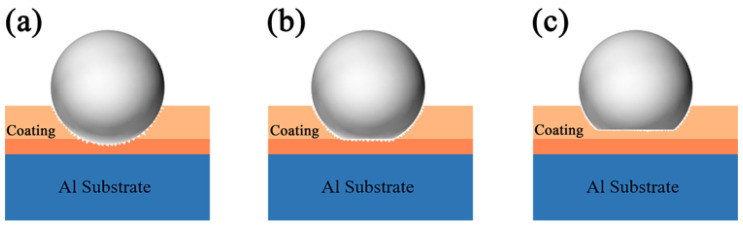
Schematic diagram of the failure mode of frictional pairs in the wear process of PEO coating; (**a**) 0 g/L, (**b**) 5 g/L, (**c**) 5 g/L + CA.

Figure 11 reveals the abrasion morphology of the PEO coating with K_2_ZrF_6_ addition. As Figure 11a demonstrates, the coating surface undergoes an obvious abrasion phase left by sliding friction. With the addition of K_2_ZrF_6_, the depth of the abrasion marks was significantly smaller compared to S1, but the width of the abrasion marks increased by 34.7% compared to S1 (Figure 11b). When citric acid was added, the abrasion mark surface of the PEO coating revealed the morphology of the original coating, showing good abrasion resistance, but its abrasion mark width was increased by 48.4% compared to S1 (Figure 11c). The reasons for this situation are shown in Figure 12. Three diagrams depict three situations that occur during frictional wear of PEO coatings: Figure 12a demonstrates that when the hardness of the friction pair (GCr15 balls with a diameter of 5 mm) is much greater than the hardness of the coating (or the coating is more sparse), the friction pair does not wear as much, and the result is a shallow, but wide, abrasion mark. When the hardness of the coating is comparable to the hardness of the friction vice (Figure 12b), wear occurs at the location where the friction vice is in contact with the coating, and the abrasion marks are relatively shallow and wider compared to S1. Figure 12c illustrates the morphology of the abrasion marks formed during wear when the coating hardness exceeds that of the friction pair, in which case the coating appears to have the shallowest and widest abrasion marks. The coatings with K_2_ZrF_6_ and citric acid showed the highest hardness and lowest wear rate, exhibiting excellent wear resistance (Table 4).

## 4. Conclusion

This study is important for the physical protection of aluminum and its alloys and improves the wear resistance of conventional plasma electrolytic oxidation coatings to a certain extent, focusing on the effect of the addition of K_2_ZrF_6_ to the PEO coatings of aluminum alloys. Given the limited solubility of K_2_ZrF_6_ in alkaline environments, citric acid was introduced to stabilize the pH value at about 8. Finally, the resulting coatings were characterized for their phase composition, microstructure, and wear resistance, yielding the following key finding:

(1) The addition of K_2_ZrF_6_ delays the appearance of soft plasma discharge and increases the thickness of the PEO coatings by 37.3% and the roughness by 27.6% at the S3 parameter compared to S1.
(2) The addition of K_2_ZrF_6_ facilitates the formation of a mullite phase and improves the densification of the PEO coating.
(3) The addition of K_2_ZrF_6_ facilitated the improvement of the breakdown voltage of the coating, which was increased by 46.0% at the S3 parameter compared with S1; it also exhibited excellent wear resistance, with a 41.8% reduction in mass wear rate compared with S1.

## Figures and Tables

**Table 1:** Adding the content of K_2_ZrF_6._

Sample	K_2_ZrF_6_ (g·L^−1^)	CA (g·L^−1^)
S1	0	0
S2	5	0
S3	5	5

**Table 2:** Parameters for tribological testing.

Friction Test Parameters	
Load	4 N
Liner speed	0.42 m/s
Time	20 min
Frictional pair, spherical	Φ 5 mm (GCr15)
Track diameter	10 mm

**Table 3:** Surface composition (at. %) of PEO coatings assessed by EDS.

Sample	Percentage of Element Content (at%)			
	Al	O	Si	Zr
S1	28.82	58.53	12.65	0.00
S2	22.63	59.46	17.84	0.08
S3	22.27	59.43	17.05	1.26

**Table 4:** Performance parameters of coatings at different negative current densities.

Sample	Thickness (μm)	Hardness (HV_0.1_)	Wear Rate (g)
S1	37.48 ± 1.7	965 ± 71.8	0.0091
S2	40.34 ± 2.2	1105 ± 73.4	0.0074
S3	51.47 ± 2.6	1195 ± 84.2	0.0053

## Data Availability

The original contributions presented in this study are included in the article material. Further inquiries can be directed to the corresponding author.
